# Built Environments and Dementia Care: Architectural Layouts as Determinants of Resident Well-Being

**DOI:** 10.1093/geroni/igaf122.1318

**Published:** 2025-12-31

**Authors:** Yifat Rom, Ido Morag, Yuval Palgi, Michal Isaacson

**Affiliations:** University of Haifa, Haifa, HaZafon, Israel; Shenkar College of Engineering and Design, Ramat Gan, HaMerkaz, Israel; University of Haifa, Haifa, HaZafon, Israel; University of Haifa, Haifa, HaZafon, Israel

## Abstract

The fast-growing number of individuals with dementia worldwide has prompted the establishment of specialized dementia units, yet these facilities may not always meet their residents’ well-being needs. This study, therefore, examined correlations between the architectural layout of seven dementia units and their residents’ well-being. Physical well-being was measured in terms of comfort and stimulation, while social well-being was measured in terms of status, behavioral confirmation, and affection. The residents’ verbal and physical agitated behaviors were also observed and documented. Diverse correlations were found between the five well-being dimensions and agitated behaviors. Negative correlations were seen between layouts that support residents’ physical well-being, verbal agitation, and all agitated behaviors combined. Yet unexpectedly, inconsistencies were seen between layouts that support residents’ social well-being and agitated behaviors, with increased agitated behaviors occurring in layouts that support status and affection. This exploratory study offers insights into the significance of the architectural layout of dementia units in augmenting residents’ well-being. Furthermore, this novel research sheds light on the potential of employing quantitative tools for comprehensively examining the spatial configurations of such units. Incorporating a descriptive well-being dimension into quantitative investigations can enhance our understanding of such environments, offering a promising avenue for optimizing residents’ overall well-being.

